# A modified Trastuzumab antibody for the immunohistochemical detection of HER-2 overexpression in breast cancer

**DOI:** 10.1038/sj.bjc.6602507

**Published:** 2005-04-05

**Authors:** G Bussolati, F Montemurro, L Righi, M Donadio, M Aglietta, A Sapino

**Affiliations:** 1 Department of Biomedical Sciences and Human Oncology, University of Turin, Torino, Italy; 2 Unit of Medical Oncology, Institute for Cancer Research and Treatment, Torino, Italy; 3 Department of Medical Oncology, San Giovanni Hospital, Torino, Italy

**Keywords:** Trastuzumab, HER-2, breast carcinoma, biotin, predictivity

## Abstract

The immunohistochemical determination of HER-2 to identify patients with advanced breast cancer candidates for Trastuzumab treatment proved neither accurate nor fully reliable, possibly because none of the current reagents detects the specific antigenic site target of Trastuzumab. To circumvent this problem, we conjugated the NH_2_ groups of Trastuzumab with biotin, and the compound obtained, designated BiotHER, was added directly to tissue sections. Biotin-labelling was revealed with horseradish peroxidase-conjugated streptavidin. Specificity and sensitivity of BiotHER immunostaining with respect to *HER-2* amplification were tested on 164 breast carcinoma samples. BiotHER staining was detected on the tumour cell membrane of 12% of all specimens and in 49% specimens with gene amplification, while absent in nonamplified tumours. Predictivity of BiotHER status with respect to the clinical outcome was analysed in 54 patients with *HER-2* amplified advanced breast cancer treated with Trastuzumab plus chemotherapy. BiotHER staining, detected in 50% of tumours with *HER-2* amplification, was an independent predictor of clinical outcome. In fact, BiotHER positivity was independently associated with increased likelihood of tumour response and reduced risk of tumour progression and death. Biotinylated Trastuzumab can thus be used for immunohistochemical detection of HER-2 overexpression in breast cancer, and has the potential to identify patients likely to benefit from Trastuzumab treatment.

Overexpression of the HER-2 transmembrane receptor is detected in 25–30% of human breast cancers (Slamon *et al*, 1987, 1989) and is a direct result of gene amplification (Pauletti *et al*, 1996) in approximately 90–95% of cases. Demonstration of HER-2 antigen overexpression in breast cancer constitutes the rationale for treatment with Trastuzumab (Baselga *et al*, 1998). Trastuzumab consists of the antigen-binding fragment (Fab) of the murine mAb 4D5, directed against the extracellular domain (ECD) of HER-2, spliced to the Fc fragment of human IgG (Baselga *et al*, 1998). The 4D5 mAb was humanised to minimise the immunogenicity associated with the murine counterpart and to maximise its potential to recruit endogenous immune effector cells (Tokuda *et al*, 1996; Baselga *et al*, 1998). As a single agent, Trastuzumab produced a response rate of 15–26% in women with HER-2-overexpressing advanced breast cancer (Cobleigh *et al*, 1999; Vogel *et al*, 2002). Combined with chemotherapy, Trastuzumab has resulted in increased response rate and survival (Slamon *et al*, 2001, Montemurro *et al*, 2004b) in this clinical setting of patients.

The clinical response to Trastuzumab can be predicted by immunohistochemical (IHC) determination of HER-2 receptor expression in tumour cell membranes (Slamon *et al*, 2001; Montemurro *et al*, 2004b). However, establishing the presence of *HER-2* gene amplification by fluorescence *in situ* hybridisation (FISH) seems to be a more accurate, reliable and cost-effective method for selecting patients eligible for Trastuzumab therapy (Elkin *et al*, 2004) than IHC, perhaps because none of the available IHC procedures detect the Trastuzumab epitope. In fact, the Trastuzumab-binding site has been mapped to the C-terminal portion of domain IV in the juxtamembrane region of the ECD (Cho *et al*, 2003). On the contrary, other commercial products used for HER-2 receptor testing do not recognise the same epitope as Trastuzumab. Specifically, Herceptest, and mAb CB11 recognise the intracellular domain of HER-2 (Corbett *et al*, 1990; Ceccarelli *et al*, 1999). On the other hand, mAb TAB250 recognises the ECD but the epitope is unknown (Ceccarelli *et al*, 1999) (Figure 1). Previously, we demonstrated that by combining the score values obtained by CB11 and TAB250 mAbs in a *double scoring system*, it was possible to predict the gene status in 58% of cases of breast cancer (Sapino *et al*, 2003), thus proving the interest for IHC detection of the ECD.

However, to predict Trastuzumab activity, it is essential to verify the availability of the Trastuzumab target epitope, especially since several studies have demonstrated the occurrence of tumours expressing variants of the HER-2 protein (Christianson *et al*, 1998; Kwong and Hung, 1998; Molina *et al*, 2001, 2002). Many cell surface transmembrane proteins, including growth factor receptors, can be released from the cell surface by a general shedding system activated by several independent mechanisms. The ECD of the HER-2 may be cleaved and shed from the receptor and can be detected in serum of 35–40% patients with metastatic breast cancer as a protein of approximately 105 kDa (Hayes *et al*, 2001). Such shedding process is actively regulated by proteolytic processes. In addition, in *in vitro* experiments using Trastuzumab as antibody for immunoprecipitation analysis, it has been shown that the soluble ECD in the medium maintains the Trastuzumab epitope, which is lost in the cell lysates (Codony-Servat *et al*, 1999). This shedding generates an NH_2_-terminally truncated HER-2 product of *M*_r_ 95 000 demonstrated in cell lines and in breast cancer tissues (Christianson *et al*, 1998; Molina *et al*, 2001, 2002). In these circumstances, patients are still selected as candidates for Trastuzumab therapy on the basis of gene amplification analyses and staining procedures that recognise the intracytoplasmic portion, but treatment is doomed to fail because of the lack of the Trastuzumab epitope.

A straightforward approach would be to use Trastuzumab as the primary Ab in IHC. However, technical reasons impede this because antigen detection using a primary Ab of the same species as the target tissue is complicated by high background staining. The present study demonstrates that, following modification, Trastuzumab can be used reliably to detect overexpression of its antigen by IHC. In a series of cancer samples of different organs, this novel IHC test was compared to commercial antibodies in detecting HER-2 overexpression and with *in situ* hybridisation procedures that reveal gene amplification. Finally, in a series of patients with *HER-2*-amplified advanced breast cancer treated with Trastuzumab plus chemotherapy, we retrospectively studied the predictivity of BiotHER status with respect to clinical outcome.

## MATERIALS AND METHODS

### Rationale for the procedure and preparation of reagents

The modification of Trastuzumab consisted in conjugating the mAb to biotin which binds NH_2_ groups of the mAb. Trastuzumab, commercially available as Herceptin® (Roche, Hertfordshire, UK), is distributed in vials for use in breast cancer treatment. One milligram of Trastuzumab from a vial of Herceptin® was diluted in saline solution (1 mg ml^−1^ concentration) and dialysed overnight in 0.1 M Na_2_CO_3_, pH 8.5. To 1 ml solution, 0.12 ml of *ε*-caproylamido-biotin-*n*-hydroxy-succinimide ester (Biospa, Milano, Italia) was added. The preparation was mixed by gentle agitation for 4 h at room temperature (RT) and dialysed in phosphate-buffered saline (PBS). The product, designated BiotHER stock solution, was stored frozen at −80°C in 20 *μ*l aliquots and sublimated. The lyophilized mAb was stored in vials.

### Immunohistochemical methods for BiotHER immunostaining

To set up the IHC procedure for BiotHER, we used cell pellet of BT474 breast cancer cells (American Type Culture Collection, Manassas, VA, USA) and a tissue array of 10 breast cancers with different *HER-2* gene status (five amplified and five nonamplified tumours). Cells were maintained at 37°C and 5% CO_2_ in Dulbecco's modified Eagle's medium 13 (DMEM) (Sigma-Aldrich) containing 10% fetal calf serum (Biochrom-Berlin). Confluent cells were scraped and centrifuged. Cell pellets were fixed in 10% neutral-buffered formalin, then embedded in paraffin. Deparaffinised tissue sections were brought to PBS, then covered with 25 *μ*l BiotHER, final dilution 1 : 2000 in PBS (protein concentration=0.5 *μ*g ml^−1^). Sections were incubated for 1 h at RT, washed twice for 5 min in PBS. Biotin label of BiotHER was then revealed incubating with horseradish peroxidase (HRP)-conjugated streptavidin diluted 1 : 50 (BioGenex, San Ramon, CA, USA) for 13 min at RT. Since preliminary tests showed that BiotHER reactivity was not improved by antigen retrieval (MW or proteinase digestion), we eventually avoided this procedure. Sections were then washed in PBS and the reaction was routinely developed in a solution of 3′-3-diaminobenzidine (DAB) and H_2_O_2_ for 5 min. Nuclei were counterstained with Mayer Haemalum for 30 s. Dehydration in alcohols, clarification in xylol and mounting in Entellan followed. Positivity corresponded to membrane staining. Evaluation was performed with the four-tiered scoring system (see below). For quality control analysis, conjugation of biotin to Trastuzumab was performed every week for a total of five biotinylation processes and the obtained BiotHER compound was tested at each time point by IHC in the control tissues. To test preservation of immunoreactivity, lyophilised BiotHER stock was tested every 2 weeks by IHC procedures on the control tissue array.

To correlate gene amplification and BiotHER immunoreactivity with Herceptest and TAB250 immunoreactivity, we tested these antibodies on tissue arrays prepared from tumours of different organs (164 primary breast carcinomas, 28 colon, 28 lung, 28 ovarian and 26 prostate cancers). All tissues were formalin fixed and paraffin embedded. In 24 breast carcinomas, immunohistochemistry using 4D5 mAb was also performed.

Herceptest (Dako Corp., Carpenteria, CA, USA) was utilised according to the manufacturer's instructions. TAB250 mAb (Zymed, San Francisco, CA, USA) was used as previously reported (Sapino *et al*, 2003). After blocking nonspecific binding sites (Histostain Plus kit, Zymed), sections were incubated for 30 min at 37°C with TAB250 diluted 1 : 40, then incubated for 20 min with secondary biotinylated anti-mouse Ig antibody (1 : 50, BioGenex), followed by HRP-conjugated streptavidin (1 : 50, StrAviGen MultiLink Kit, BioGenex) for additional 20 min at RT. Sections were also stained using the 4D5 mAb (not commercially available and kindly supplied by Dr PG Natali, Regina Elena Cancer Institute, Rome, Italy). For IHC, mAb 4D5 was diluted 1 : 1000 following the methods used for TAB250 mAb. As with BiotHER, we did not observe any advantages for reactivity of 4D5 by antigen retrieval procedures. The four-tiered scoring system (0, 1+, 2+ and 3+ score values) suggested by the manufacturers to evaluate the results of Herceptest and TAB250 in breast cancer tissue was applied to these Abs and also to BiotHER and 4D5 results. HER-2 protein expression was defined as negative (scores 0 and 1+) or positive (scores 2+ and 3+). This analysis was performed double-blindly.

All cases were then studied with Chromogenic Insitu Hybridization (CISH) and/or FISH to assess *HER-2* gene amplification. BiotHER sensitivity and specificity were calculated using the known *HER-2* gene status of the tumour as the gold standard.

### Fluorescence *in situ* hybridisation or CISH procedures

PathVysion *HER-2*/*neu* probe kit (Vysis Inc., Downers Grove, IL, USA) was used for FISH analysis. In brief, sections were baked overnight at 56°C, and invasive carcinoma components were selected based on haematoxylin and eosin-stained sections, deparaffinised in CitriSolv, dehydrated in 100% ethanol and air-dried. Slides were then treated with proteases for 45–60 min, denatured and hybridised overnight at 37°C with the probes (*HER-2*/*neu*/CEP17 SG probe 35–171060, Vysis Inc., Downers Grove, IL, USA). Slides were washed with posthybridisation buffer at 72°C, counterstained with 4′,6′-diamidino-2-phenylindole (DAPI), mounted and stored in the dark prior to signal enumeration. Slides were first scanned at low power with a DAPI filter to identify areas of optimal tissue digestion and nonoverlapping nuclei. Cases were scored as amplified when the ratio of HER-2/chromosome 17 signals were ⩾2.0. This analysis was performed double-blindly. For CISH analysis, the sections were deparaffinised in xylene and 99% alcohol, air-dried for 10 min and then heated above 98°C for 15 min in CISH Tissue Heat Pretreatment. The sections were then rinsed in dH_2_O and digested with pepsin for 10 min. Afterwards, sections were washed in dH_2_O, dehydrated with graded alcohol, and air-dried. The ready-to-use digoxigenin-labelled *HER-2/neu* probe (consisting of two contig BAC clones; Zymed Lab) was applied onto slides, which were covered by 14 × 14 mm coverslips (10 *μ*l probe mixture/slide). The slides were denatured on a hot plate (94–95°C) for 5 min, and the hybridisation was performed overnight at 37°C. After hybridisation, the slides were washed with 0.5 × SCC prewarmed at 75°C for 5 min, followed by three washes in dH_2_O. The *HER-2*/*neu* probe was detected with sequential incubations with mouse anti-digoxigenin antibody for 45 min followed by incubation with polymerised HRP-anti-mouse antibody for another 45 min and diaminobenzidine according to the manufacturer's instructions (Zymed). Tissue sections were lightly counterstained with methyl green.

Amplified cases had both low level amplification (showing 6–10 signals per nucleus in >50% of cancer cells, or a small gene copy cluster), and high level *HER-2* gene amplification (showing a large gene copy cluster in >50% of carcinoma cells or >10 separate gene copies), as defined in the original report (Sapino *et al*, 2003).

### Patients

To evaluate the impact of BiotHER immunostaining on Trastuzumab response, IHC analysis with BiotHER was performed in specimens from 54 women with *HER-2* amplified advanced breast cancer. These cases were selected because: (i) they had been treated with Trastuzumab combined with chemotherapy, (ii) their tumour blocks were available for retesting and (iii) their follow-up data was available. All 54 cases were re-evaluated with BiotHER and FISH. The patients had started treatment between September 1999 and July 2004. Immunohistochemical positivity was originally scored 3+ in 45 patients and 2+ in nine patients. For seven of these 2+ tumours, a FISH test showing *HER-2* amplification had been obtained before initiating therapy with Trastuzumab.

#### Treatment

In all 54 patients, Trastuzumab was administered using the weekly schedule (4 mg kg^−1^ loading dose, followed by 2 mg kg^−1^ weekly). Trastuzumab was combined with docetaxel 75 mg m^−2^ every 3 weeks in 42 patients, including 34 who were treated in a phase II multi-institutional trial (Montemurro *et al*, 2004a), and vinorelbine 30 mg m^−2^ on days 1 and 8 every 3 weeks for the remaining 12 patients. For both docetaxel and vinorelbine, the treatment was planned to be given for a maximum of six cycles with concomitant weekly Trastuzumab. Patients showing tumour response or stabilisation upon the completion of chemotherapy continued weekly Trastuzumab until disease progression or unacceptable toxicity.

Tumour response was classified according to WHO criteria (CR: complete remission, PR: partial remission, SD: stable disease, PD: progressive disease) (Miller *et al*, 1981). Response rate (RR, CR plus PR) is reported together with its 95% confidence interval (95% CI), as calculated by the Wilson's method (Wilson, 1927). The Kaplan–Meier product limit method was used to determine: (1) time to progression (TTP), which was calculated from the date of the first dose of docetaxel and Trastuzumab and the date of the first documented tumour progression or death in the absence of tumour progression; (2) overall survival (OS), which was calculated from the date of the first dose of Trastuzumab to the date of documented death of the patients. Alive patients were censored at the date of most recent follow-up visit.

#### Statistical analysis

The concordance among IHC assay methods was studied by the K statistics. The difference in RR between subgroups of patients was analysed by Fisher's Exact Test. Differences in TTP and OS between different subgroups of patients were analysed by the log-rank test. The effect of BiotHER positivity on RR was studied by logistic regression analysis including potential covariates. Results are reported as odds ratio (OR) with 95% CI. Similarly, the effect of BiotHER positivity on TTP and OS was studied in a Cox's Proportional Hazards model including potential covariates. Results are reported as hazard ratio (HR) with 95% CI. The *P*-values were calculated by means of the Wald statistic and considered significant if ⩽0.05. The statistical analysis was performed using the SPSS-PC software, version 11.5 (SPSS, Chicago, IL, USA).

## RESULTS

### Definition of HER-2 expression by immunohistochemistry

**Figure 2:**
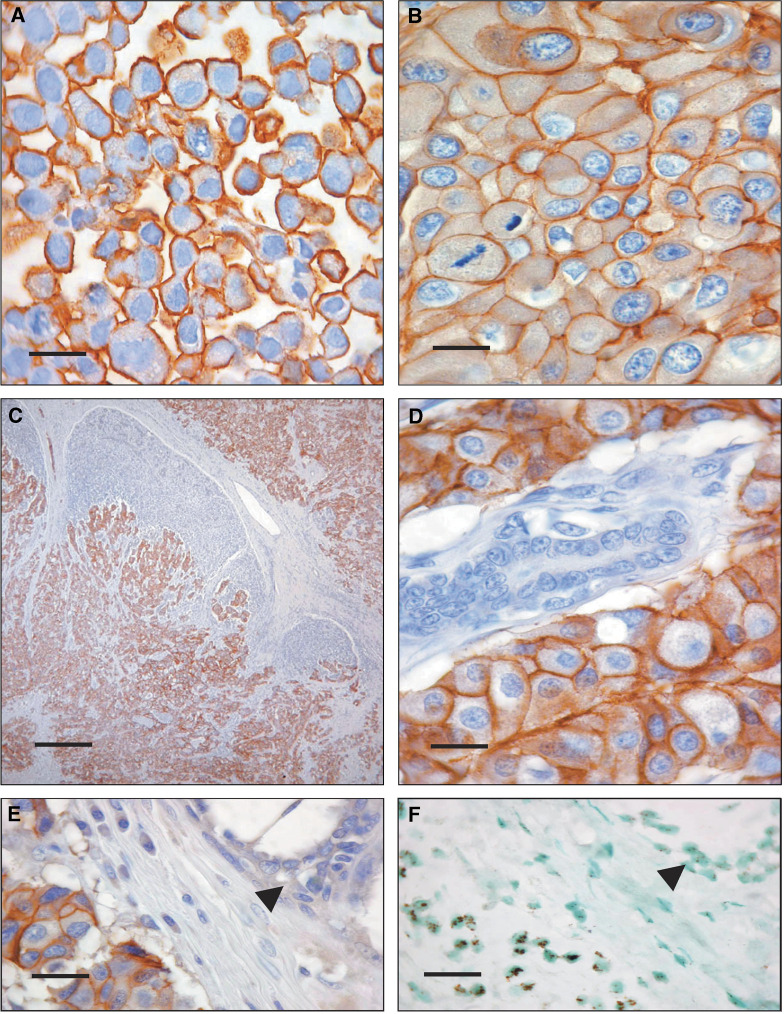
BiotHER immunostaining is restricted to membranes of (**A**) BT474 cells and of (**B**) cancer cells in paraffin-embedded tumour samples; (**C**) no background or (**D**) normal cell staining is observed. (**E**) BiotHER stains amplified tumour cells (**F**) (CISH; large gene copy cluster/nucleus), and (**E, F** arrows) is negative in normal nonamplified residual cells (two signals/nucleus).

HER-2 protein receptor overexpression was located on membranes of BT474 breast cancer cells with all the mAbs tested, that is, Herceptest, TAB250 and BiotHER (Figure 2A). The quality control analysis demonstrated that conjugation process with biotin had a reproducibility of 100%, while the immunoreactivity was lost after 3 months from biotinylation. Staining with BiotHER was invariably detected on the cell membrane, while cytoplasmic staining (Figure 2B) or staining of stroma and of normal residual epithelial cells was never observed (Figure 2C, D). Fluorescence *in situ* hybridisation analysis of the 164 specimens from primary breast cancers showed *HER-2* gene amplification in 42 cases (26%). The only 21 specimens that stained positively by BiotHER had *HER-2* gene amplification (Figure 2E, F arrows). Concordance of BiotHER with Herceptest and TAB250 are summarised in Table 1a and b. MAb 4D5 was studied in 24 of the 164 breast carcinomas. Of 14 cases with *HER-2* amplification, nine were positive with both BiotHER and 4D5, whereas two were negative with both tests, and in three cases 4D5 alone was positive. The 10 nonamplified cases studied were negative for both 4D5 and BiotHER staining (*K* for overall concordance=0.75, *P*<0.01).

None of the other specimens from different cancers (see Materials and Methods) showed *HER-2* gene amplification or BiotHER staining, except for two cases of colon cancer. Both cases were *HER-2* gene amplified, BiotHER positive and TAB250 positive. Herceptest was positive in only one of these cases and negative in the other.

### BiotHER immunostaining and clinical outcome in patients receiving Trastuzumab for advanced breast cancer

Of the 54 tumour specimens from patients selected for the clinical outcome analysis, BiotHER was negative (score 0 or 1+) in 27 cases and positive (score 2+ or 3+) in the remaining 27 cases (50%). The original tissue specimens of this case series derived from different laboratories and were probably prepared using different fixation methods. Seven of these specimens showed unspecific immunostaining (cytoplasm and/or background staining and no clear membrane staining) with BiotHER and were thus ultimately considered negative.

**Figure 3:**
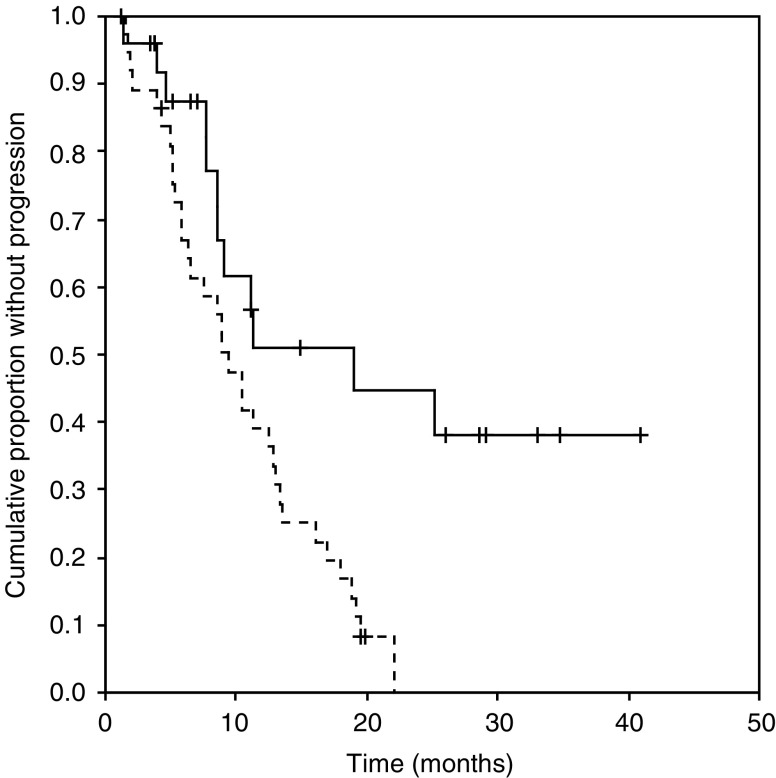
Kaplan–Meier estimates of time to progression (TTP) according to BiotHER status. The solid line represents patients with BiotHER-positive tumours and the dashed line patients with BiotHER-negative tumours.

**Figure 4:**
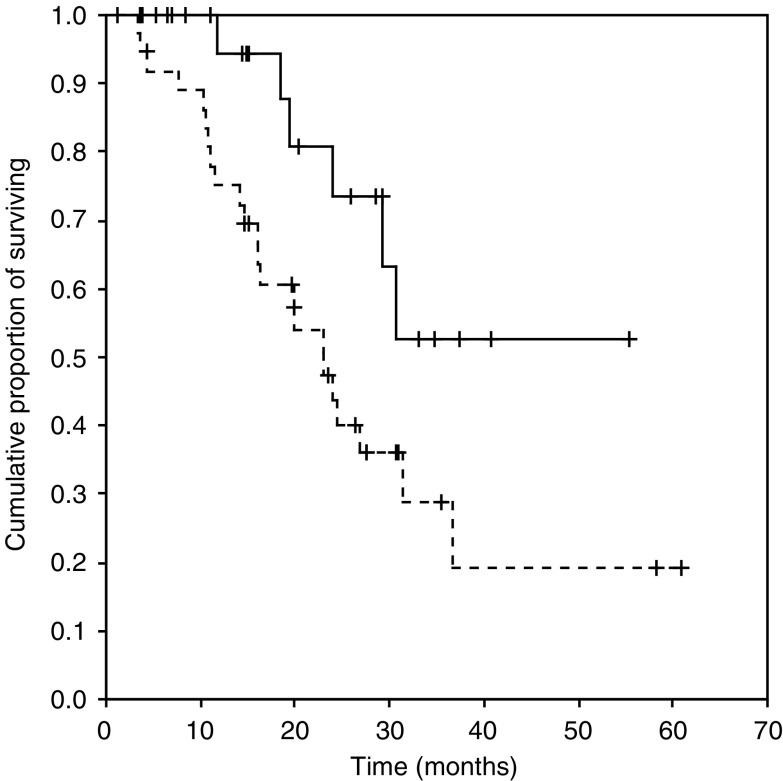
Kaplan–Meier estimates of overall survival (OS) according to BiotHER status. The solid line represents patients with BiotHER-positive tumours and the dashed line patients with BiotHER-negative tumours.

At the time of this analysis, 36 patients have progressed and 22 patients have died. The median follow-up for patients who were still alive at the last follow-up contact was 22 months (range 4–58 months). Overall, the combination of chemotherapy plus Trastuzumab produced a 74% RR (95% CI 61–84%). The Kaplan–Meier estimates of median TTP for the overall population was 11 months (95% CI 8–14 months, Figure 3). The median overall survival was not reached (Figure 4). The 2-year survival was 55% (95% CI 39–71%).

None of the differences seen in the distribution of clinical characteristics between patients with BiotHER-positive and -negative tumours approached statistical significance (Table 2). The RR was significantly higher in BiotHER-positive patients (89%, 95% CI 72–96%), compared to BiotHER-negative patients (59%, 95% CI 41–75%) (Table 3).

BiotHER positivity was associated with longer median TTP (19 *vs* 9 months for patients with BiotHER-positive and -negative tumours, respectively, *P*=0.03) and median OS (not reached *vs* 23 months for BiotHER-positive and -negative tumours, respectively, *P*=0.04) (Figures 3 and 4).

The effect of BiotHER positivity on RR, TTP and OS was studied in multivariate analysis models including other variables, which were found to be significantly associated with the outcomes in the univariate analysis (data not shown). BiotHER positivity was independently associated with increased likelihood of tumour response (Table 4). Of the other potential covariates, only prior exposure to chemotherapy for metastatic disease was independently associated with reduced response rate. Survival analysis showed that BiotHER status was the only independent predictor for both OS and PFS. BiotHER positivity was associated with reduced risk of death (HR 0.391, 95% CI 0.152–1.003, *P*=0.05) and tumour progression (HR 0.04, 95% CI 0.213–0.912, *P*=0.03).

## DISCUSSION

In the present work, we devised a simple strategy, based on the biotinylation of Trastuzumab (BiotHER), which allows the use of the anti-HER-2 humanised Ab for IHC. Thus, BiotHER can be used as a primary Ab when evaluating the availability of Trastuzumab-specific binding sites in breast tumour tissue sections. We demonstrated that BiotHER immunoreactivity was restricted to tumours with *HER-2* gene amplification. In addition, BiotHER positivity was a strong predictor of clinical outcome in patients with advanced breast cancer treated with Trastuzumab and chemotherapy.

Trastuzumab is a human-murine chimeric mAb, which retains only the small antigen-binding fragment of the murine 4D5 immunoglobulin (Carter *et al*, 1992) that specifically recognises the ECD of HER-2. In clinical trials, to screen patients for enrolment, immunostaining with 4D5 mAb was used but only in combination with other Abs (Mass *et al*, 2001; Slamon *et al*, 2001; Bookman *et al*, 2003) because of concerns that use of 4D5 mAb alone might underestimate HER-2 detection in formalin-fixed and paraffin-embedded tissues. The fact that only a subset of FISH-amplified tumours scored positively by 4D5, as well as by BiotHER in the present study, may be explained by the shedding of ECD activated by proteolitic processes. In fact, it has been shown that 22.4% of breast cancer tissues express only the p95 NH_2_-truncated form of HER-2 (Christianson *et al*, 1998), owing to shedding of ECD. The possible truncation of HER-2 with loss of ECD prompted us to use Trastuzumab as the primary Ab in IHC procedures, in order to select patients whose tumour cells maintain the target epitope. In addition, 4D5 is not commercially available. HER-2 diagnostic testing recommendations (Bilous *et al*, 2003), proposed by different nations, do not consider 4D5 mAb as an IHC marker and, to our knowledge, no work has been published on the correlation between 4D5 immunostaining and *HER-2* gene amplification by FISH analysis in tissue specimens. We decided upon biotinylation of Trastuzumab to avoid incubation with a secondary anti-human IgG, which could create problems of background staining. In addition to reducing the IHC procedure to a single step (no secondary antibody needed), BiotHER immunoreaction does not require antigen retrieval. In properly-fixed tissue samples, we observed that: (i) the IHC reaction with BiotHER was limited to the plasma membrane of tumour cells; (ii) no cytoplasmic staining was observed and (iii) normal epithelial or stromal cells did not react whatsoever. However, in the series of patients treated with Trastuzumab, we noted suboptimal (i.e., cytoplasmic and background) BiotHER staining in seven cases (three tumours were nonresponsive and four responded to Trastuzumab treatment) received from external laboratories. These data confirm the need for optimal tissue processing as a prerequisite for reliable HER-2 IHC staining (Bilous *et al*, 2003). Comparison of the reactivity of BiotHER with that of other commercially available Abs such as Herceptest and TAB250 demonstrated a low concordance.

Not surprisingly, we observed instead an 87.5% agreement of the results obtained with BiotHER and mAb 4D5; while 12.5% of IHC reactivity with mAb 4D5 was not confirmed with BiotHER. The reason for this minor discrepancy is not clear since Trastuzumab has a high affinity for the HER-2 ECD (*K*_d_=5 × 10^−9^ M) although we cannot exclude that a slight structural variation of the 4D5 Fab occurred after humanisation. In fact, the crystal structure of mAb 4D5 demonstrates that the complementarity determining region 3, which determines the antibody–antigen interaction, is composed of six highly flexible hypervariable loops (Zhang *et al*, 1999).

When we calculated the specificity of BiotHER reactivity using the known *HER-2* gene status of the tumour as the gold standard, we observed that none of the unamplified samples were BiotHER- or 4D5-positive. In addition, differently from Herceptest and TAB250, all cases positive with BiotHER even those scored as 2+ showed invariably high level of gene amplification by FISH analysis (50% sensitivity) and all cases scored as 1+ or 0 were nonamplified (100% specificity).

The second part of our study evaluated the clinical impact of the results of BiotHER IHC staining. For this purpose, predictivity of BiotHER results with respect to RR, TTP and OS was retrospectively studied in a series of 54 patients with *HER-2* amplified advanced breast cancer who had received Trastuzumab plus chemotherapy. Patients whose tumours was BiotHER positive had superior RR, TTP and OS, compared with patients with BiotHER-negative tumours. BiotHER positivity was an independent predictor of clinical outcome in both the overall and the amplified population. We thus suggest that BiotHER identifies the subset of patients with *HER-2* amplified tumours achieving the highest benefit from Trastuzumab-based therapy. Obviously, these results should be interpreted with caution and require prospective confirmation for two main reasons: (1) the analysis included a small number of subjects, (2) in the amplified population, the response rate in patients with BiotHER-negative tumours was 59%, a rate that does not exclude a possible, even if less pronounced, benefit from the addition of Trastuzumab to chemotherapy also in this subset of patients. On the other hand, our findings provide a possible explanation for the low activity rate of single agent Trastuzumab in patients selected on the basis of conventional IHC testing and FISH. As stated before, the ECD of HER-2, which represents the accessible target for immunotherapy with Trastuzumab, can be shed (Hayes *et al*, 2001) and splice variants of *HER-2* with deletion in ECD exons can occur (Christianson *et al*, 1998; Kwong and Hung, 1998; Molina *et al*, 2002). Another explanation to the response of patients with negative BiotHER immunoreaction is that mechanisms of HER-2 shedding might change under Trastuzumab therapy and chemotherapy, with less of the HER-2 protein fragment being released to the serum, which would make the c-erbB-2-positive tumour cells a better target for anti-HER-2 antibody treatment (Luftner *et al*, 1999; Molina *et al*, 2001).

Both FISH testing and Abs that are directed against the intracellular domain, such as Herceptest and CB11, or against undefined ECD epitopes, such as TAB250, do not account for the possible absence of the Trastuzumab epitope on the cell surface.

In conclusion, our results suggest that, in patients with breast cancer and *HER-2* gene amplification, the use of BiotHER as primary Ab in IHC procedures has the potential to further improve the definition of the target population for Trastuzumab-based treatment. Furthermore, we provide the first evidence that mAbs humanised for use in therapy (an increasingly popular approach) can be used in IHC, simultaneously revealing target availability and forecasting clinical efficacy.

## Figures and Tables

**Figure 1:**
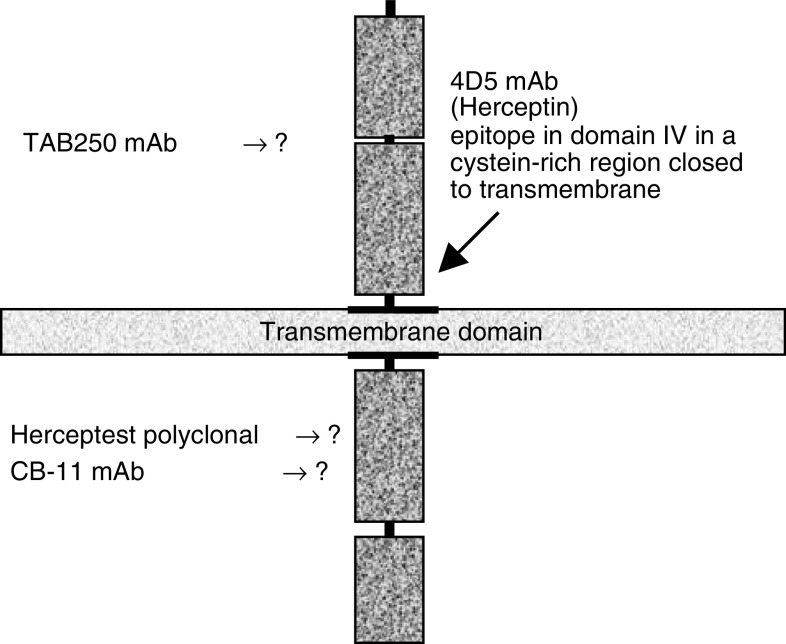
Site of binding of different antibodies used in the IHC detection of HER-2 overexpression in breast cancer. The exact epitope recognised by some reagents is presently unknown.

**Table 1:** Concordance of BiotHER with (a) HercepTest, (b) TAB250

**BiotHER score**	**0–1**	**2–3**	**Total No. of specimens**
(a)			
0–1	85	58	143
2–3	1	20	21

(b)			
0–1	109	34	143
2–3	0	21	21

*Note*: *K*=0.45, *P* <0.01.

**Table 2:** Patients' demographics according to BiotHER status

	**BiotHER−**	**BiotHER+**
**Characteristic**	***N*=27**	***N*=27**
Median age in years (range)	55 (35–75)	50 (36–76)
Median DFS in months (range)	21 (0–134)	24 (0–119)
Median number of metastatic sites (range)	2 (1–4)	2 (1–3)
Patients with single metastatic sites	8 (30%)	11 (41%)
Visceral involvement (liver+lung)	19 (70%)	22 (81%)
Liver involvement	14 (52%)	15 (56%)
Prior chemotherapy for metastatic disease	18 (67%)	23 (85%)
Prior adjuvant/neoadjuvant chemotherapy	15 (56%)	18 (67%)
Prior exposure to antracyclines	18 (67%)	16 (59%)

N=number; DFS=disease-free survival from initial diagnosis to metastatic progression.

**Table 3:** Response to Trastuzumab-based therapy according to BiotHER status

**Response**	**BiotHER pos.**	**Rate (%)^a^**	**BiotHER neg.**	**Rate (%)**
CR+PR	24	89^b^	16	59
CR	2	7	4	15
PR	22	81	12	44
SD	2	7	9	33
PD	1	4	2	7

a Because of rounding, the sum of percentages is not always=100.

b Fisher's exact test for the difference in the rate of responders (CR+PR), *P*=0.03.

CR=complete remission; PR=partial remission; SD=stable disease ; PD=progressive disease.

**Table 4:** Logistic regression analysis of tumour response

	**Univariate**			**Multivariate**		
**Variable**	**OR**	**95% CI**	***P***	**OR**	**95% CI**	***P***
BiotHER positivity	5.500	1.323–22.862	0.04	4.589	1.042–20.207	0.04
Prior chemotherapy for metastases	0.176	0.045–0.688	0.01	0.216	0.052–0.906	0.04

OR=odds ratio; CI=confidence interval.
